# Honor in the Wild: Virtuous Violence between the Hobbesian Trap and Social Order

**DOI:** 10.1007/s12110-023-09455-1

**Published:** 2023-09-06

**Authors:** Michael Windzio, Dirk Baier

**Affiliations:** 1 University of Bremen, 330440, 28334 Bremen, PF Germany; 2 ZHAW Zurich University of Applied Sciences, Winterthur, Switzerland

**Keywords:** Honor, Deterrence, School violence, Smith-Price model, Social context, Multilevel analysis

## Abstract

“Culture of honor” means that individuals deter others by signaling their commitment to violent retaliation. We develop a multilevel explanation of cross-level interdependence of honor and violence. According to our concept of *system-level honor*, a social system is loaded with deterrence signaling if culture of honor is highly prevalent in the system. In line with the Smith and Price (1973, in *Nature,* https://www.nature.com/articles/246015a0) model, we argue that high system-level honor discourages Prober-Retaliator behavior: some individuals might tend to challenge others they assume to be inferior to increase their own reputation. Both individual culture of honor and system-level honor contribute to an increase in violence (H1; H2). However, as system-level honor and deterrence become more prevalent, the impact of individual honor diminishes because engaging in violent behavior becomes increasingly expensive within such a system (H3). As a second contextual effect, inequality in culture of honor should therefore increase violent behavior because it encourages Prober-Retaliator behavior (H4). We analyze the effect of culture of honor on school violence among 15-year-old adolescents. Disentangling the micro- and context-level effects of culture of honor on violent behavior in a multilevel analysis framework allows the estimation of a cross-level interaction using a large data set from more than 25,000 adolescents in more than 1,300 schoolroom contexts. Results are in line with our H3, but not with H4. Model-based predictions show that the deterrent effect must be unrealistically high to generate an equilibrium of average violence.

People do not value a social organization that involves intense and widespread violence. Nevertheless, in the absence of institutionalized third parties, the potential for violence remains a constant threat both within and between groups (Richerson & Boyd, 2006:226–27). We will call this type of situation as the “wild” (Bicchieri, 2017), but the wild did not exist only in human prehistory. It exists today in deprived urban neighbourhoods (Anderson, 2000), in prisons (Black, 1998:76), in fragile states (Acemoglu & Robinson, 2019:98–99), and in encounters among adolescents in schools (Windzio & Baier, 2009).

Honor, reputation of strength, and deterrence by signaling commitment to retaliate (Cohen & Vandello, 2001) often correspond to self-governance within and between communities. Research in anthropology, criminology, and urban sociology indicates that nonviolent social interaction is unstable in “honor cultures” (Basáñez, 2016) due to the “Hobbesian trap” (Pinker, 2011): if coercive power is not centralized by third parties, actors must rely on self-help (Black, 1998:38). Most actors want to avoid violent encounters and deter others by signaling their commitment to retaliate violently against any kind of assault. At best, the Hobbesian trap creates a situation of peace for the moment that easily turns into a self-reinforcing escalation of reciprocal violence (Pinker, 2011:34).

In our study, we develop a multilevel model of a cross-level interdependence of honor and violence. Based on our model, deterrence occurs when individuals signal a culture of honor, which is measured by their personal commitment to respond violently, even if they themselves prefer nonviolent approaches. We apply the term “system-level honor” when a culture of honor is highly prevalent in a social system, i.e., when many individuals strive for honor and respect by signaling their commitment to violent retaliation. According to our argument, system-level honor may moderate the individual effect of culture of honor on violent behavior. Although individual culture of honor (“individual honor”) and high system-level honor both *increase* violence, the effect of individual honor *decreases* with increasing system-level honor because proactive violent behavior becomes extremely costly the more the system is loaded with system-level honor.

Previous models assume that the degree of *inequality in signaling* also has an independent effect. Since it pays off for individuals to gain a reputation regarding their level of strength, some individuals might tend to challenge others to increase their own reputation. In the field of evolutionary biology, this behavior is referred to as the “Prober-Retaliator” strategy (Smith & Price, 1973). It is effective only when the distribution of deterrence within the social system is unequal, creating a reasonable chance of encountering potential inferiors who can be challenged. We formalize these arguments on *system-level honor* and *inequality in signaling* and test them empirically in a multilevel framework using data from more than 25,000 adolescents in more than 1,300 school classes.

## Theory and Research

Whereas Thomas Hobbes highlighted humans’ selfishness and predatory tendencies, Jean-Jacques Rousseau described humans as inherently gentle, peaceful, altruistic, and noncompetitive (Pinker, 2011:33–36; Sapolsky, 2018:305). Recent studies suggest, however, that both perspectives are partially justified (Wrangham, 2019). Humans are prosocial creatures when interacting with close relatives but also with strangers if they identify them as members of their own cultural group (Richerson & Boyd, 2006:221–25). Self-domestication came along with the emergence of self-control capacities (Hare & Woods, 2020), allowing us to reduce *reactive* aggression. At the same time, however, our capacity to exert *proactive* aggression has increased due to the evolution of self-control (Wrangham, 2019:164–66). Reactive aggression is a response to a perceived threat and driven by emotions, whereas proactive aggression “involves the purposeful attack with an external or internal reward as a goal, rather than an effort to remove the source of fear or threat” (Wrangham, 2019:28). Self-control helped our ancestors to collaborate in ostracizing or even executing bullies, alphas, and noncooperative cheaters within their own group (Boehm, 2011:163) also by applying *proactive* violence (Wrangham, 2019:246).

### Honor and Virtuous Violence

The distinction between reactive and proactive aggression closely relates to the concept of *virtuous violence* (Fiske & Rai, 2015). Virtuous violence is a means to regulate social relationships. In the social dimension of *authority ranking*, for instance, violence can reestablish a social hierarchy. This is “probably the most common moral motivation for violence” (Fiske & Rai, 2015:24). According to the virtuous violence concept, violence is not necessarily immoral. Durkheim had already emphasized that people consider murder and theft as ideal types of immoral acts but may kill in a retaliatory manner because honor demands them to do so (Durkheim, 1999:156, 164). Engaging in proactive violence or signaling a strong commitment to violent retaliation can be viewed as rational strategies to enhance or maintain one’s social status.

The role of reputation, or honor, as a form of social integration (Thrasher & Handfield, 2018) has been illustrated in a study on Greek Sarakatsan shepherds. Showing strength in acts of violent retaliation increases a man’s honor: “Although aimless violence is dishonorable there is no missing the pleasure it gives when a man is forced to kill; nor the prestige which it brings him” (Campbell, 1974:318). A man’s reputation for manliness deters others from touching his family or his property. The worst insult is to insinuate “shameless” sexual behavior by a man’s daughter, sister, or mother (Campbell, 1974:269–71). Following from this, virtuous violence is an essential element of culture of honor, in which violence is sometimes even regarded as a moral duty (Bicchieri, 2017:32).

### Ecologies of Culture of Honor: No Centralized Coercive Power

Culture of honor thrives in contexts where a centralized monopoly of power does not exist or is weak, at best (Nisbett & Cohen, 1996). Culture of honor consists of signals of strength and deterrence, which was a dominant mechanism of social order during the major part of global human history (Henrich, 2020). Still today, commitment to culture of honor is a signal to deter others from assaulting one’s property, status, and integrity (Daly & Wilson, 1988:129; Cohen & Vandello, 2001).

In her study on Mediterranean pastoralists, Jane Schneider described how ecology, agricultural and pastoral economies, and weak states correspond to honor and violence as mechanisms of social order (Schneider, 1971). Retaliation and ostracism are also major issues illustrated in Christopher Boehm’s study on blood revenge in Montenegrin mountain villages. Boehm argues that his subjects did not regard killing for revenge as a violation of moral imperatives. Contrariwise, “it was a moral necessity that a man (or a clan) take vengeance, if a decent social status was to be maintained” (Boehm, 1984:66).

The issue of honor, violence, and masculinity as a mechanism of social order was the focus in a seminal study by cultural psychologists Nisbett and Cohen (1996). European settlers established herding economies in the (US) South. Since a “herdsman continually faces the possibility of losing his animals through the actions of others,” the “issue of protection is, therefore, a very serious one, and the herdsman cultivates an acquaintance with violence and weapons to deter those who would prey on him” (Nisbett & Cohen, 1996:89).

Culture of honor emerges when legitimate monopoly of coercive power and legitimate official law are absent (Anderson, 2000:317; Boehm, 1984; Cohen & Vandello, 2001:164; Nisbett & Cohen, 1996). It is a way to avoid a permanent *bellum omnium contra omnes* when there is no disinterested third party (Black, 1998:98) or “bystander” who enforces the conflicting parties to act nonviolently (Pinker, 2011:35). According to the commitment model (Frank, 1988; Nesse, 2001), signaling commitment to violent retaliation in such a “wild” situation is a way to influence others’ behavior (Schelling, 2001). For instance, during the Greek war against the Persians 2,400 years ago, Xenophon placed his troops in a position where retreat was impossible, signaling to the Persians that defeating them was their only option (Schelling, 2001). This apparent commitment altered the psychology of the battle to the Greeks’ advantage because the Persians did have an option to retreat. Likewise, committed retaliators signal, according to the commitment model, that they deliberately eliminate certain options, in this case the option of not retaliating. Bluffing and faking can also be strategies of increasing one’s status (Cohen & Vandello, 2001:174–75), but such strategies become costly in contexts consisting of high shares of committed retaliators. Without a third party, which is the *Leviathan* in Hobbes’s political philosophy, peace is always unstable due to the Hobbesian trap. “The key to the deterrence policy, though, is the credibility of the threat that you will retaliate,” which provides “an explanation of the incentive to invade for trifles: a word, a smile, and any other sign of undervalue” (Pinker, 2011:34). It doesn’t come as a big surprise that homicide rates in non-state societies, during our Neolithic past, were disturbingly high (Bowles, 2009; Gat, 2008; Keeley, 1996; Kelly, 2000), although this issue is still debated in anthropology (Allen & Jones, 2016; Fry, 2015; Gat, 2015, 2019).

A modern ecology of culture of honor exists in “total institutions” (Goffman, 1990), where the social order among inmates resembles anarchic forms of bottom-up self-organization (Black, 1998:82). In large US prisons, gangs organized along ethnic and racial boundaries create social order by governance systems based on reputation of strength, signaling commitment to violent retaliation and rigid norms (Skarbek, 2014:82–87), sometimes codified in written rules (Skarbek, 2014:91). Self-organization is possible because prison guards are unable to supervise and sanction all transactions among inmates (Skarbek, 2014:20). Some guards even exchange deliberate ignorance for inmates’ compliance (Sykes, 1971:57; Windzio, 2006:344). Unmonitored niches where inmates’ own social order can flourish are thus widespread in most custody institutions, but such niches also exist in schools. Some scholars advance the view that in the US, “school violence is at pandemic proportions” (Osborne, 2004:147). Violence in schools is a significant issue in Germany, although the precise figures differ somewhat. In both the 2014 and 2018 surveys of “Health Behavior in School-Aged Children,” Germany was below the average for all countries in terms of prevalence. Eight percent of male and two percent of female 15-year-old adolescents reported having been involved in a physical fight in the past 12 months in 2018 (World Health Organization, 2020:107). A large survey in the German state of Lower Saxony reported much higher rates of physical violence in schools (Bergmann et al., 2019:46): nearly 20% of 15-year-old adolescents were victims of physical violence in the school context in the past 6 months (17.9%). This proportion slightly increased compared with two years earlier (17.2%). Other studies provide even higher figures. More than 25% of 10- to 12-year-old pupils in Germany said they had been beaten at school at least once in the past month (Rees & Main, 2015:79). Given these results, violence is definitely an important issue in most German schools. In our study, we conduct an empirical analysis of schools as environments where surveillance is not absent, yet interpersonal violence remains prevalent. This violence arises due to a self-organized social order among peers in unsupervised niches.

### Individual and System-Level Honor

Given the Hobbesian trap (Pinker, 2011:34), how does deterrence moderate individuals’ violent behavior? Deterrence results from signaling commitment to violent retaliation (Schelling, 2001). An individual’s culture of honor is an indicator of this commitment. Yet, deterrence also results from the composition of culture of honor in the respective social system, in our case, the schoolroom. We call this *system-level honor*, which is the degree of deterrence at the level of the social system. In line with existing research (Enzmann et al., 2004; Windzio & Baier, 2009), higher levels of *individual* honor might increase the probability of violent behavior. At the social system level of the classroom, in contrast, high system-level honor might ceteris paribus decrease the effect of individual honor on violence if the costs exceed the benefit, which is likely when the system is loaded with signals of commitment to violent retaliation. Theoretically, high system-level honor could even decrease the effect of individual honor so strongly that it returns the overall prevalence of violence back to the overall mean value—which we would regard as an equilibrium (see below).

Assume we have two actors, an offender *i* and a retaliator *j*. The aim of retaliation is to maintain the reputation of strength and to avoid signs of weakness. As a result of capitulating, *j* would lose his reputation of strength and status (Thrasher & Handfield, 2018:375). The absence of signaling a commitment to retaliation might encourage other potential offenders to target and compete for the resources possessed by individual *j*. Moreover, rational actors can apply proactive violence in order to increase their status. According to evolutionary biology (Smith & Price, 1973), most violent conflicts within the same species are neither lethal nor do they cause serious injury. Smith and Price conducted computer simulations to compare the payoffs of different strategies. They examined strategies referred to as Hawk, which consistently employs harmful weapons, Mouse, which never does, and Retaliator, which initially adopts nondangerous behavior but responds with harm when faced with opponents’ harmful behavior. Playing Retaliator rather than Hawk turned out to be an evolutionarily stable strategy (ESS) (Smith & Price, 1973:16), which means “a strategy such that, if most of the members of the population adopt it, there is no ‘mutant’ strategy that would give higher reproductive fitness” (Smith & Price, 1973:15). Another strategy was Prober-Retaliator, which has a high probability to be harmless but sometimes probes the opponent with a harmful strategy. He switches back to the harmless strategy as the opponent retaliates but remains harmful if the opponent acts harmlessly. Contrary to the Retaliator, the Prober-Retaliator exerts proactive violence to increase his status but is not committed to the harmful strategy. According to the model, shares of Retaliators and Prober-Retaliators increase in the population, but the relative share between the two depends on the share of Mice in the respective social system. The reason is that probing is only beneficial when playing against Mice (Smith & Price, 1973:16). The relative payoff for a strategy thus depends on the composition of the social system. If it is composed of a high share of harmless strategies, the average payoff of violent behavior is high. In contrast, higher shares of committed retaliators who violently respond to violent behavior decrease the payoff. Therefore, refraining from Prober-Retaliator behavior may be a sensible strategy where high levels of system-level honor exist.

### System-Level Honor and Prober-Retaliator Behavior in the Wild

Unpopular norms, such as virtuous violence and revenge, emerge and persist because actors try to derive “clues as to what it is appropriate to do in a given setting” (Bicchieri, 2006:180). Given a high prevalence of a *descriptive norm* (Bicchieri, 2006:29; Cialdini et al., 1990) of violence, which refers to the normalcy of violence, it is costly to display commitment to nonviolence. Signs of weakness encourage others to offend because successful offenders increase their own reputation in the respective social system.

Elijah Anderson (2000) made a similar observation in his study on the “codes of the street”: it is beneficial in terms of status for actors to strive for acknowledgement in their peer group by searching for potential victims on whom they can demonstrate their boldness. Thus, “physicality is a fairly common way of asserting oneself” (Anderson, 2000:68). When successfully challenging somebody, street-oriented children increase their social status (Ernst & Lenkewitz, 2020). Decent families, in contrast, try to socialize their children by teaching them social norms and respect towards authorities. Accordingly, a high prevalence of “decent” children may increase the probability of violence in their community because Prober-Retaliators may find an opportunity for intimidation and violent acts (Anderson, 2000:100).

The central argument in our study is that proactive violence aimed at improving one’s status becomes extremely costly when the social system is heavily deterred, meaning that the honor at the system level is high. Using violence “rationally” as an instrument to increase one’s status becomes more unlikely in the face of committed retaliators who are prepared to impose high costs on proactive violence. In contexts characterized by high system-level honor, individuals who possess a strong individual culture of honor and would typically adopt Prober-Retaliator strategies to elevate their status might now be inclined to avoid violent behavior. This shift is influenced by the fact that most of their opponents signal a defense, indicating a well-protected position. Assuredly, there is still a lot of violence if system-level honor is high because of signaling error and “the high incentive to invade for trifles” (Pinker, 2011:34). Resulting from signaling error, violence evolves “behind the backs” of actors. Proactive, “rational” prober-retaliator-violence aiming at increasing one’s status declines due to the increasing share of committed retaliators, and the decreasing share of Mice or “decent kids,” respectively. In other words, the effect of *individual* commitment to violent retaliation might decline if the anticipated negative consequences (costs) of proactive violence become extremely high.

Violence *v*_*ij*_ due to high levels of individual honor *h*_*i*_ occurs when the utility of violence *U*(*v*_*i*_) outweighs its costs *C*(*v*_*i*_). According to our argument, the prevalence of violence is increasing among subjects who agree with the culture of honor. With higher levels of system-level honor due to high shares of committed retaliators in the system, however, the probability of meeting another committed retaliator increases, which discourages Prober-Retaliators. The system-level honor (*sh*) in system *j* generates extra costs *C*(*sh*_*j*_) of violence, depending on the probability *p* of meeting a committed retaliator *j*, which might reduce the effect of individual culture of honor on violence.

Yet a further aspect of social composition can encourage violence, as Smith and Price (1973) demonstrated in their simulation models: given the average level of honor and its variance, the probability of an unequal matching might become higher the more unequal the distribution of honor is. Unequal matching encourages Prober-Retaliators to challenge nonviolent actors, such as Smith and Price’s Mice. At the context level of the respective social system, the probability of violence might increase with increasing inequality in honor. Therefore, the probability of an encounter of actors with different levels of individual honor *p*_*j*_{*h*_*j*_* ≠ h*_*i*_} might have a positive effect on violent behavior *v*_*ij*_ of individual *i* in context *j*.

1 $$v_{ij}=\left\{\begin{array}{c}0\;\text{if}\;U\left(v_i\right)-C\left(v_i\right)-p_j\left\{C\left({sh}_j\right)\right\}+p_j\left\{h_j\neq h_i\right\}\leq0\\1\;\text{if}\;U\left(v_i\right)-C\left(v_i\right)-p_j\left\{C\left({sh}_j\right)\right\}+p_j\left\{h_j\neq h_i\right\}>0\end{array}\right.$$

System-level honor *sh*_*j*_ is measured as the mean value of culture of honor in context *j*, and *p*_*j*_{*h*_*j*_* ≠ h*_*i*_} is the standard deviation (instead of the variance because of unequal classroom sizes) of all honor *h*_*ij*_ in system *j*. It measures the inequality of the distribution of culture of honor, so

2 $$\begin{array}{ccc}{p}_{j}\left\{{h}_{i}\ne {h}_{j}\right\}=\sqrt{\frac{\sum {\left({h}_{ij}-{\overline{h} }_{ij}\right)}^{2}}{{n}_{j}}}& \mathrm{ and}& {p}_{j}\left\{C\left({sh}_{j}\right)\right\}=\frac{\sum {h}_{ij}}{{n}_{j}}\end{array}$$

We draw upon the concept of “norms in the wild,” as introduced by Christina Bicchieri. This term is applicable to our study because we do not analyze the relationship between honor norms and violence through laboratory experiments. Instead, we rely on survey data collected from real-life contexts, aligning with the methodology proposed by Bicchieri (2017:91). In addition, we agree with the anthropological and sociological literature that honor norms—as signals of commitment to violent retaliation—emerge when institutions of social control are weak or absent. This is “the wild,” where individuals, families, and clans regulate conflicts on their own since they cannot rely on police, rule of law, and law courts. Since the costs of violence strongly increase with increasing system-level honor, it becomes less “rational” to apply violence proactively with the objective of increasing one’s status. Empirically analyzing such processes requires one to decompose the effects of culture of honor on violent behavior into an individual-level and a system-level (context) component, which we can do by using multilevel analysis, testing four hypotheses derived from our model:

- Hypothesis 1: the higher the individual level of deterrence (honor), the higher the probability to be a violent perpetrator.
- Hypothesis 2: the higher the average level of deterrence (honor) in a social system, which we call system-level honor, the higher an individual’s probability to be a violent perpetrator.
- Hypothesis 3: at higher levels of the social system level of deterrence (honor), however, the effect of individual deterrence (honor) decreases because costs increase.
- Hypothesis 4: the more unequal the distribution of deterrence (honor) in a respective social system, the higher the probability of violent behavior because Prober-Retaliators have a higher probability of encountering nonviolent opponents.

A recent study distinguished between “revenge” and “purification” (Thrasher & Handfield, 2018), which is similar to Saplosky’s distinction between “honor as self-reliance” (Sapolsky, 2018:285) and “violence turned inward” (Sapolsky, 2018:288). We follow this distinction in our own study and rephrase it as “honor and external violence” and “honor and internal dominance” (Enzmann et al., 2004). We focus on the *external* subdimension of culture of honor, indicated by “violence-legitimizing norms of masculinity” (VLMN), but rerun our models also for the *internal* subdimension (see Tables 1 and 2 here, and Table 3 in the Appendix) for a robustness check.

**Table 1:** Culture of honor as a determinant of school violence. Logistic multilevel models, odds ratios

	(1)	(2)	(3)	(4)
	external	external	internal	internal
boy = 1, other = 0	3.658^***^	3.659^***^	4.080^***^	4.080^***^
*Culture of honor*				
honor (VLNM)	2.212^***^	2.234^***^	1.883^***^	1.888^***^
mean VLNM class	2.001^***^	1.937^***^	2.235^***^	2.210^***^
VLNM * Mean VLNM class	0.571^***^	0.610^***^	0.507^*^	0.523^***^
SD VLNM class	1.225	1.314	1.144	1.156
VLNM * SD VLNM class	1.287	—	1.045	—
var(_cons[klcode])	0.189***	0.190***	0.217***	0.217***
var(VLMN[klcode])	0.020	0.020	0.016	0.015
cov(cons/VLMN)	− 0.029	− 0.030	− 0.058*	− 0.058*
R^2^ McKelvey & Zavoina	0.2053	0.2055	0.1674	0.1675
*N* (School classes)	1365	1365	1365	1365
*N* (Students)	26284	26284	26271	26271

Exponentiated coefficients ^*^ *p* < 0.05, ^**^ *p* < 0.01, ^***^ *p* < 0.001.

Source: KFN School Survey, authors’ computation.

**Table 2:** Culture of honor as a determinant of school violence. Logistic multilevel models, odds ratios

	(1)	(2)
	external	internal
boy = 1, other = 0	3.600^***^	3.824^***^
*Culture of honor*		
honor (VLNM)	1.738^***^	1.532^***^
mean VLNM class	1.362^*^	1.046
VLNM * mean VLNM class	0.614^***^	0.613^**^
SD VLNM class	1.356	1.206
*Individual-level controls*		
Age (years)	1.023	1.038
German	ref	ref
former Soviet Union	0.936	1.004
Turkish	1.024	1.067
Polish	1.229^*^	1.294^*^
Other	0.901	0.931
Parents: high secondary/university	1.036	1.019
Parents: unemployed/social assistance	1.189^*^	1.201^*^
Grade in Math, German	0.873^***^	0.848^***^
Low secondary	ref	ref
Medium secondary	0.805^*^	0.779^**^
Integrated	0.846	0.809^*^
High secondary	0.558^***^	0.510^***^
No violence in childhood	ref	ref
Some violence in childhood	1.564^***^	1.590^***^
Often violence in childhood	1.988^***^	2.056^***^
Risk-seeking (low self-control)	1.603^***^	1.735^***^
*Classroom-level controls*		
mean risk-seeking (low self-control)	0.883	1.010
mean grades in class	1.151	1.137
var(_cons[School-class])	0.178^***^	0.193^***^
var(VLMN)	0.022	0.050
cov(_cons/VLMN)	− 0.014	− 0.041
R^2^ McKelvey&Zavoina (RIM)	0.2504	0.2366
*N* (School classes)	1365	1365
*N* (Students)	26,284	26,271

Exponentiated coefficients: ^*^ *p* < 0.05, ^**^ *p* < 0.01, ^***^ *p* < 0.001.

Source: KFN School Survey, authors’ computation.

## Methods

### Sample

In 2013, 2015, and 2017, representative self-report surveys were conducted among adolescents (average age 15 years) in the German state of Lower Saxony (Bergmann et al., 2019), whose ministry of education approved the survey. School classes were randomly drawn (stratified sampling by school type) from all classes in the respective school year (special schools with a focus other than learning were excluded). We surveyed 9,512 adolescents in 2013 (response rate: 64.4%), 10,638 adolescents in 2015 (response rate: 68.5%), and 8,938 in 2017 (response rate: 59.2%). Taken together, a maximum of 26,284 pupils are included in our analyses, attending a total of 1,365 classes varying in size between 10 and 34 pupils (see Table 4 in the Appendix for descriptive statistics). Parents gave written consent for their child to participate. Before the survey started, we made respondents aware of data privacy regulations. The written survey took an average of 90 min and was supervised by trained test administrators, usually in the presence of the teacher.

### Measurements

#### Dependent variable

We measure physical violence at school in the preceding semester with two items: “I intentionally hit or kicked another student” and “I extorted another student and forced them to give me money or things” (ranging from 1 = never to 6 = several times per week). Perpetrators are pupils who performed at least one of the two behaviors “one or two times.” Accordingly, the dependent variable is dichotomous (0 = no perpetrator of school violence, 1 = perpetrator of school violence). The mean of the dependent variable is 0.16 (*n* = 26,284).

#### Independent variables

We measure violence legitimizing norms of masculinity (VLMN) based on Nisbett and Cohen’s (1996) concept of “culture of honor.” Our measurement applies two scales developed by Enzmann et al. (2004).

*External culture of honor* consists of five items: “A man should be willing to defend his wife and children with violence,” “A man who is unwilling to defend himself against insults with violence is a weakling,” “Men should be allowed to own firearms to protect their family or property,” “A real man is willing to strike if someone speaks ill of his family,” and “A real man is strong and protective of his family.” Reliability is adequate as shown by McDonald’s ω = 0.73 and Cronbach’s α = 0.72. The items could be agreed to from “1 = not true” to “4 = exactly true.” We computed the mean over the set of items and further transformed it into a grand mean centered scale with mean of 0 and a standard deviation of 0.60 (*n* = 26,284).

*Internal culture of honor* consists of three items: “A man as the head of the family must be obeyed by his wife and children,” “If a wife cheats on her husband, the husband is allowed to beat her,” and “The husband is the head of the family and is allowed to assert himself by force if necessary.” Reliability is rather low with McDonald’s ω = 0.59 and Cronbach’s α = 0.53, but at any rate we focus our interpretation on the external subdimension. Again, we computed the mean over the set of items and further transformed it into a grand mean centered scale with a mean of 0 and a standard deviation (SD) of 0.47. The correlation between external and internal culture of honor, *r*, is 0.48.

#### Control variables

*Age* is one of the control variables (13–19 years, mean = 14.87, SD = 0.71). In terms of the country of birth and nationality of the biological parents and of the respondent, 76% were of German origin; 7% came from countries of the former Soviet Union, 4% from Turkey, 3% from Poland, and 10% from other countries. 49% of the respondents’ parents have a high level of education (high school or university). 5% of our students attend a low secondary; 23%, a medium secondary; 37%, an integrated; and 35%, a high secondary school. At least one parent is currently unemployed or receiving social assistance in 6% of the families. In addition, the following three variables were considered:

*Grades* the mean value of the grades of the school subjects German and Mathematics in the most recent school report was calculated (6 = very good, 1 = insufficient; *r* = 0.40, mean = 0.01, SD = 0.78 [centered]).

*Parental violence in childhood* separately for father and mother, adolescents were asked to report whether they had carried out various forms of violence in childhood (before age 12). The items were: “smacked,” “grabbed/pushed hard,” “threw object,” “hit with object,” “hit/kicked with fist,” and “punched/beaten up” (response options: 1 = never to 6 = several times a week). The items were summarized to an index: experienced (almost) no parental violence in childhood (84%), sometimes (regularly in 1 or 2 dimensions) (35%), and often (regularly at least in 3 dimensions) (11%).

*Low self-control* we used the “risk seeking” subscale by Arneklev et al. (1993:230) to measure low self-control. The measurement of self-control is required to empirically test the “general theory of crime” (Gottfredson & Hirschi, 1990), which is one of the most prominent theories in criminology. The scale consists of the following four items: “I like to test my limits by doing something dangerous,” “I like to take risks simply because it’s fun,” “Sometimes I find it exciting to do things that can put me in danger,” and “Excitement and adventure are more important to me than safety.” Items could be agreed to from “1 = not true” to “4 = true exactly.” We took the mean of the set of items but did not mean-center the resulting scale (McDonald’s ω = 0.82, Cronbach’s α = 0.81, mean = 2.21, SD = 0.77). Table 5 in the Appendix shows the pairwise correlations between the scales.

### Analytic Strategy

Rather than being a “nuisance,” the clustered nature of the data is an “interesting phenomenon” in our study (Snijders & Bosker, 2012:8). The multilevel structure allows the aggregation of individual-level information to the context level of the classroom. By computing the mean value of culture of honor in each classroom as an indicator of system-level honor, we decompose the effect of culture of honor in multilevel logistic regression models (Snijders & Bosker, 2012, chap. 17) into an individual-level and a context-level component. In addition, the classroom-specific standard deviation indicates the degree of inequality in signaling. Since our main hypothesis focuses on the interaction between individual-level culture of honor and systemic (schoolroom)-level culture of honor, we center both variables around their grand means and estimate a cross-level interaction: first, a random slope allows the effect of individual-level culture of honor to vary across contexts (classrooms). Second, we estimate the cross-level interaction to test our hypothesis that the slope of the individual-level effect of culture of honor on violence decreases with increasing classroom-level, system-level honor. We visualize the interaction terms using average marginal effects over a set of values of the moderator variable, and by plotting predicted probabilities.

## Results

Table 1 shows four multilevel logistic regression models predicting violent behavior in schools. In models 2 and 4, the interaction term between individual-level honor (VLMN) and its cross-level interaction with the classroom standard deviation of VLMN (SD VLMN class) was removed from the equation since the effect is far from being significant. Aside from this, results do not change considerably, which is why we interpret models 2 and 4 only. The main explanatory variable in models 2 and 4 is honor (VLMN), which is our indicator of commitment to violent retaliation. Model 2 shows that the odds of violent behavior are increased by factor 3.659*** for boys relative to girls. Moreover, a one-unit increase in honor (VLMN) increases the odds of violence by factor 2.234***. At the context level of the classroom, a one-unit increase in the mean value of honor (VLMN class) (= system-level honor) increases the odds by factor 1.973***. In contrast, the cross-level interaction between the individual- and the system-level honor is significantly negative since the odds ratio of 0.610*** is far below 1. Results are similar in model 4, with honor (VLMN) (internal) as the main predictor. In line with our theoretical considerations, system-level honor increases the odds of violent behavior but also moderates the effect of individual honor: the higher the system-level honor, the smaller the effect at the individual level. In contrast to our hypothesis, however, we find no effect of inequality in culture of honor on violent behavior. Following our argument, this effect should have been a result of an increasing probability to meet Mice the more unequal the distribution of culture of honor is, given its average level in the respective social system. Accordingly, the increasing level of systemic honor already discourages Prober-Retaliator behavior, so that there is no additional effect *p*_*j*_{*h*_*j*_* ≠ h*_*i*_} (Eq. 2) of inequality in deterrence in the respective social system.

Models 1 and 2 in Table 2 enhance the equation with important confounders. Nevertheless, the basic pattern in Table 2 remains robust: while the main effects of honor (VLMN) are positive, the cross-level interactions are negative (0.614*** and 0.613**), although the class-level main effect of the mean value of honor (VLMN class) is insignificant in model 2 (internal). Hence, the main effects tend to be positive, whereas the negative interaction term suggests declining individual-level effects, depending on the mean level of VLMN in the classroom. We described this characteristic as an indicator of how strongly the social system is loaded with deterrent signals of commitment to violent retaliation. Since the interpretation of interaction terms is challenging for nonlinear models, however, we will come back to these effects below.

Although categories of immigrant origin do not systematically explain violent behavior in school, the only effect in model 2, Table 2, is of Polish, but any explanation would be beyond the scope of our study. Conditional on the control variables, unemployment or social assistance of parents significantly increases the odds of violence. Moreover, the better the grade-point-average in mathematics and German, the lower the odds of violence, and the higher the educational level in the stratified German education system, the more the odds tend to decrease. In line with previous studies (Windzio & Baier, 2009), propensity toward violent behavior increases if students suffer from parental violence during childhood. Finally, the higher the value in the risk-seeking dimension of self-control, the higher the odds of violent behavior. The fit of the full models in Table 2 is at least satisfactory (R^2^ > 0.23), and the reduced models in Table 1 show an R^2^ value of > 0.16.

**Fig. 1:**
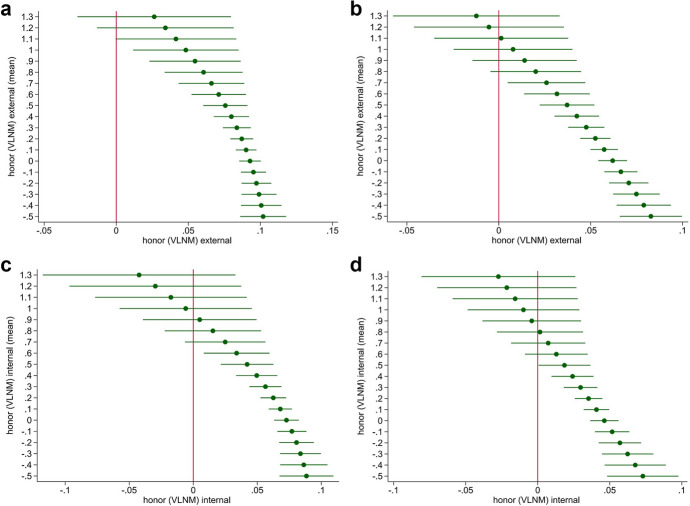
VLNM at the class level moderates individual-level VLNM. Average marginal effects of interaction terms. (A) Boys and girls, external, Model 2, Table 1, reduced; (B) Boys and girls, external, Model 1, Table 2, full; (C) Boys and girls, internal, Model 4, Table 2, reduced; (D) Boys and girls, internal, Model 4, Table 2, full. Source: KFN School Survey, authors’ computation

Understanding the combined influence of the main effect and cross-level interaction term is easier if we predict average marginal effects (AMEs). An important advantage of AMEs is that they show changes in probabilities instead of odds ratios (Long, 1997:74). Our theoretical argument in the previous section suggested that as system-level honor increases, the costs associated with signaling violence at the individual level also increase. Consequently, we posited that the impact of individual honor on violent behavior should decrease as system-level honor increases within a school class. Figure 1 shows cross-level interaction effects for honor (VLNM) for boys and girls. On the left side we show predictions of the effects from the reduced models in Table 1, and on the right-side, predictions from the full models in Table 2. In line with our expectations, there is a strong and positive effect of individual-level culture of honor on violent behavior. However, the effect declines with increasing level of average culture of honor in the respective social system—which we called *system-level honor*. High system-level honor strongly increases the costs of Prober-Retaliator violent behavior because the probability of an encounter with a committed Retaliator increases. In our view, this result corroborates the distinction between reactive and proactive violence. If reactive violence were the primary driving force behind violence in schools, the interaction effect would be positive. This means that it would amplify the positive main effects since each violent encounter would trigger a self-reinforcing cycle of violence and reactive violent responses. Contrariwise, we can conclude from our results that, in line with our model, proactive Prober-Retaliator violence decreases in social contexts with high system-level honor.

**Fig. 2:**
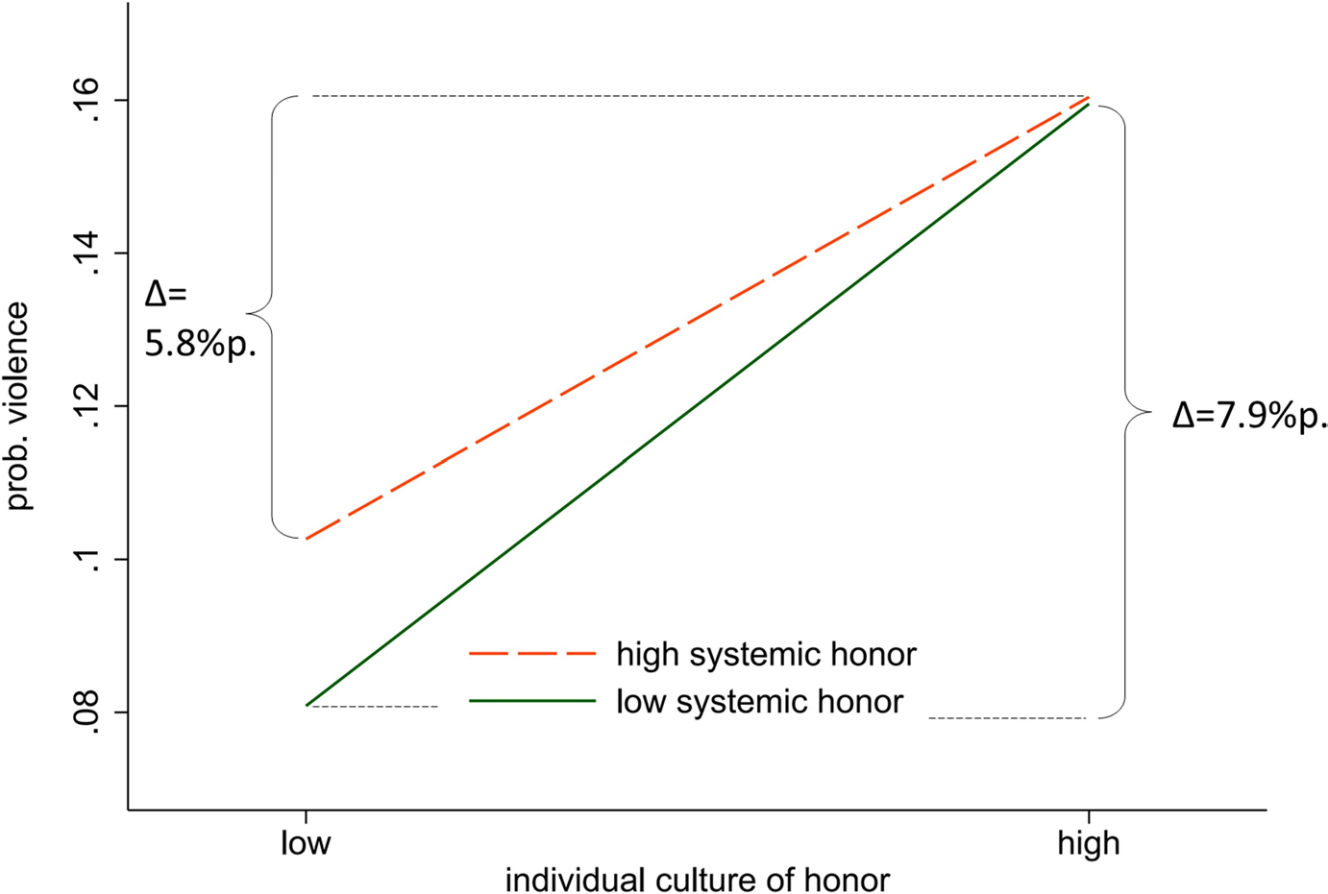
Interaction effect of high and low system-level honor (external) on the probability of violence, predictions from Model 1 in Table 2. Source: KFN School Survey, authors’ computation

Another way of illustrating the interaction effect is the conditional effect plot in Fig. 2, here for individual- and system-level honor (external) and the probability of violence. High and low values of honor are defined by one standard deviation above and below the mean. The solid line represents the effect of individual culture of honor in a schoolroom with *low systemic honor*. The dashed line represents the effect of individual-level culture of honor in a situation of *high systemic honor*—a high average value of culture of honor in a classroom. The solid line shows a steeper increase in the probability of violent behavior. In other words, the difference in slopes represents the *strength* of the interaction effect. When system-level honor is low (solid line), a change in individual-level honor from one standard deviation (SD) below the mean to one SD above the mean generates a change in the probability of violence of 7.9 percentage points. At a high level of system-level honor (dashed line), in contrast, the same change in individual-level honor corresponds to a change in the probability of violence of 5.8 percentage points. In other words, at a high level of system-level honor, the individual-level effect is only ~ 73% of the effect at a low level of system-level honor (0.058 / 0.079*100).

Presenting differences in probabilities in this way also clarifies that the decreasing influence of individual-level culture of honor with increasing system-level honor does *not* result from a ceiling effect or from “a regression to the mean” effect because the overall mean probability of violence (0.16) is far below the natural limit of 1. Despite a high potential for even much higher probabilities, the strength of the individual-level effect nevertheless declines with increasing system-level honor. Of course, causal interpretations of results from most cross-sectional studies are problematic. Other approaches include fixed effects models that only use the variation of the dependent variable between classes within the same schools. By taking the selectivity of students into particular schools into account, such models better capture causal effects (Ernst & Lenkewitz, 2020). On the other hand, our approach to using random-coefficient multilevel models enables us to explicitly account for classroom explanatory variables. The survey design of our study is based on a random sample of school classes, and not schools, so the fixed effects approach is not feasible.

Our sample consists of more than 1,300 school classes, resulting in a substantial variance in both contextual factors and individual behaviors. Moreover, we capture the individual- and context-level effect of culture of honor simultaneously in our model, which is why we do not think that selectivity of students is a severe problem. Finally, we controlled for many confounders in Table 2, such as school type, low self-control, parental violence, education, and ethnic origin. Most of these confounders are risk factors of violent behavior as well as correlates of VLMN (Windzio & Baier, 2009), resulting in an empirical test which is rather conservative. As a result, the interaction effect turns insignificant for boys (“external” VLMN) and girls (“internal” VLMN) when we ran the full models separately for girls and boys (Table 3 in the Appendix), although the basic pattern remains the same.

**Fig. 3:**
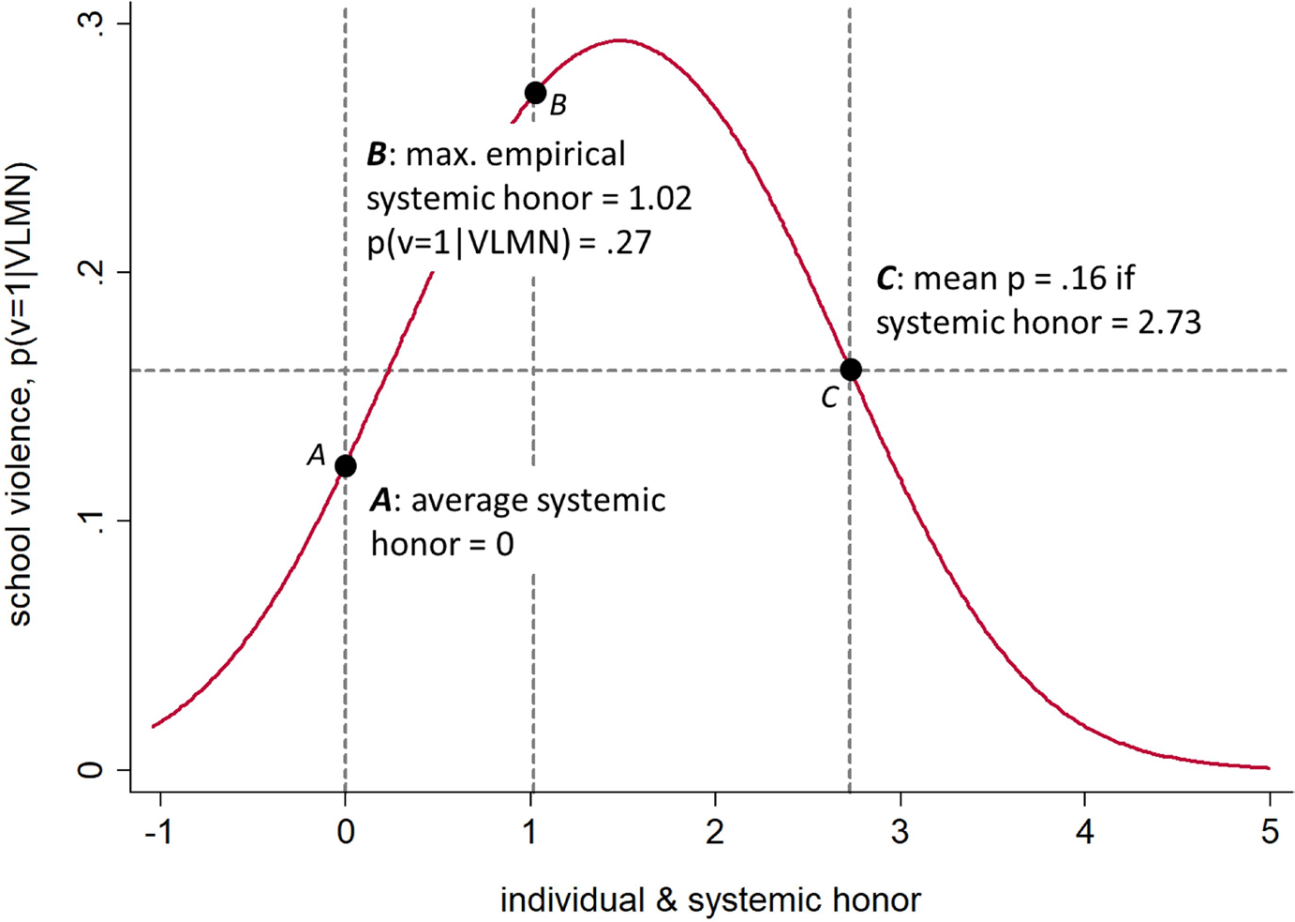
Probability of violent behavior over values of system-level honor, based on Model 1, Table 1. Source: KFN School Survey, authors’ computation

Figure 3 shows predicted probabilities of violence conditional on VLMN from the logistic multilevel Model 2 in Table 1, which we use because the gross effects best capture the classroom situation “as is” for the average individual. Predicted probabilities result from a parallel increase in individual and system-level honor. Recall that the main effects increase violence, but the interaction term (0.610***) decreases the main effects. Point A in Fig. 3 shows the average level of (mean centered) systemic honor (= 0). If both system-level and individual honor increase to the maximum empirical value of system-level honor at point B (= 1.02, see Table 4 in the Appendix), the probability of school violence would increase to 0.27. As deterrent effects that would lead to an equilibrium, however, both individual and system-level honor would have to increase to 2.73. Only then would deterrence reestablish the *average* level of school violence of 16% (Point C). Yet such a high value of deterrence is far out of the range of the empirical values (see Table 4). Accordingly, even though a high prevalence of committed retaliators decreases the effect of individual honor, this kind of deterrence does *not* necessarily lead to an average or even nonviolent equilibrium in real, empirical settings.

## Conclusion

Prehistoric humans did not suffer from a permanent *bellum omnium contra omnes*. However, the fact that groups have often established institutionalized practices of conflict resolution (Boehm, 2015) indicates a problem to be solved. In addition to institutions of peacemaking (Fry, 2015), *cultures of honor* were an attempt at preventing violence. In cultures of honor, actors signal their commitment to retaliate violently. They try to deter potential offenders by signaling their commitment to impose high costs to the offender. As a result, it is likely that the prevalence of violence within and between groups was high during the Pleistocene (Bowles, 2009; Choi & Bowles, 2007). In the wild, norms of honor “perform important governance functions in societies with weak mechanisms for organizing and controlling endogenous violence” (Thrasher & Handfield, 2018:372).

Yet “the wild” is not limited to prehistoric humans, or to present-day social groups living in more or less stateless environments or failed states (Acemoglu & Robinson, 2019). If many actors in the respective social system are, on average, extremely inclined to respond violently “for trifles” (Pinker, 2011:34), an unintended dynamic of tit-for-tat violence could emerge, which is difficult to stop without relying on a powerful third party. “The wild” also exists in custody institutions, among gangs of adolescents in deprived neighborhoods, and in unsupervised niches in schools. We tested hypotheses derived from our theoretical model on how the context-level situation of system-level honor moderates the effect of individual-level culture of honor on violence in schools. Starting from anthropological research on virtuous violence and culture of honor, we argued in line with the Smith and Price (1973) model that the social composition of the context can moderate the effect of individual culture of honor on violence. According to our theoretical model, the expected costs of violent Prober-Retaliator behavior rise with increasing system-level honor, thereby violence declines. High shares of committed retaliators already seem to be a sufficient condition to discourage Prober-Retaliator behavior. Actors with a given level of culture of honor thus behave differently depending on the social composition of their context with respect to the costs of system-level honor *C*(*sh*_*j*_). Theoretically, deterrence due to system-level honor can reduce the effect of individual honor on violence so strongly that it returns violence back to its mean value. Predictions from our model have shown, however, that this relative equilibrium is far out of the range of the empirical value of system-level honor. Consequently, we should not hope that deterrence reduces violence in the real, empirical world of secondary schools.

In our view, the decomposition of the culture-of-honor effect into an individual- and context-level component is an important contribution and could be applicable to settings such as custody institutions, deprived neighborhoods, schools, fragile states, and even to war and peace in the global world system.

## Data Availability

Stata do-file and data are available on request from the corresponding author.

## Appendix

Tables 3, 4 and 5

**Table 3:** Culture of honor as a determinant of school violence. Logistic multilevel models, odds ratios

	(1)	(2)	(3)	(4)
	external boys	internal boys	external girls	internal girls
*Culture of Honor*				
honor (VLNM)	1.706^***^	1.571^***^	2.009^***^	1.333^*^
mean VLNM class	1.181	0.912	1.672	1.556
VLNM * mean VLNM class	0.822	0.679^*^	0.337^***^	0.649
SD VLNM class	1.191	1.363	1.793	0.743
Age (years)	1.030	1.049	1.020	1.034
German	ref	ref	ref	ref
former Soviet Union	0.945	0.994	0.938	1.075
Turkish	0.889	0.918	1.280	1.410^*^
Polish	1.011	1.053	1.685^**^	1.829^***^
Other	0.868	0.881	0.998	1.094
Parents: high secondary/university	1.079	1.060	0.910	0.890
Parents: unemployed/social assistance	1.195	1.198	1.121	1.146
Grade in Math, German	0.851^***^	0.829^***^	0.945	0.918
Low secondary	ref	ref	ref	ref
Medium secondary	0.963	0.922	0.520^***^	0.494^***^
Integrated	0.988	0.939	0.582^***^	0.548^***^
High secondary	0.677^**^	0.627^***^	0.341^***^	0.288^***^
No violence in childhood	ref	ref	ref	ref
Some violence in childhood	1.470^***^	1.493^***^	1.784^***^	1.856^***^
Often violence in childhood	2.012^***^	2.080^***^	1.925^***^	1.993^***^
Risk-seeking (low self-control)	1.551^***^	1.674^***^	1.759^***^	1.911^***^
*Class-level controls*				
mean risk-seeking (low self-control)	0.805	0.869	1.098	1.458
mean grades in class	1.207	1.196	1.039	0.993
var(School class)	0.175***	0.174***	0.393***	0.402***
var(VLMN)	0.050	0.006	0.025	0.280
cov(_cons/VLMN)	− 0.022	− 0.011	− 0.065	− 0.155
R^2^ McKelvey&Zavoina (RIM)	0.1146	0.1024	0.1918	0.1515
*N* (Classrooms)	1358	1358	1360	1360
*N* (Students)	12,902	12,894	13,382	13,377

Exponentiated coefficients: ^*^ *p* < 0.05, ^**^ *p* < 0.01, ^***^ *p* < 0.001.

**Table 4:** Descriptive Statistics, *N* = 26,284

	Mean	SD	Min	Max
*N* students in class (Model 1, Table 2)	21.74	4.52	10	34
School violence	0.16	—	0	1
			*N* = 22,101	*N* = 4,183
boy = 1, other = 0	0.49	—	0	1
Age (years)	14.87	0.71	13	19
former Soviet Union	0.07	—	0	1
Turkish	0.04	—	0	1
Polish	0.03	—	0	1
Other	0.10	—	0	1
Parents: high secondary/university	0.49	—	0	1
Parents: unemployed/social assistance	0.06	—	0	1
Grade in Math, German	0.01	0.78	− 2.92	2.08
Medium secondary	0.23	—	0	1
Integrated	0.37	—	0	1
High secondary	0.35	—	0	1
Some violence in childhood	0.11	—	0	1
Often violence in childhood	0.05	—	0	1
Risk-seeking (low self-control)	2.21	0.77	1	4
mean risk-seeking (low self-control)	2.21	0.21	1.59	3.03
honor (VLNM), external	0	0.6	− 1.04	1.96
mean VLNM external class	0	0.22	− 0.58	1.02
SD VLNM external class	0	0.12	− 0.31	0.37
honor (VLNM), internal	0	0.47	− 0.38	2.62
mean VLNM internal class	0	0.16	− 0.32	0.94
SD VLNM internal class	0	0.15	− 0.3	0.68

**Table 5:** Correlations of scales, *N* = 26,284

	Honor (VLNM), external	Mean VLNM external class	SD VLNM external class	Risk-seeking (low self-control)	Mean risk-seeking (low self-control)
Honor (VLNM), external	1				
Mean VLNM, external	0.36***	1			
SD VLNM, external	0.20***	0.56***	1		
Risk-seeking (low self-control)	0.33***	0.11***	0.08***	1	
Mean risk-seeking (low self-control)	0.15***	0.42***	0.29***	0.27***	1
